# Cancer care at the time of the fourth industrial revolution: an insight to healthcare professionals’ perspectives on cancer care and artificial intelligence

**DOI:** 10.1186/s13014-023-02351-z

**Published:** 2023-10-09

**Authors:** Iman Hesso, Reem Kayyali, Debbie-Rose Dolton, Kwanyoung Joo, Lithin Zacharias, Andreas Charalambous, Maria Lavdaniti, Evangelia Stalika, Tarek Ajami, Wanda Acampa, Jasmina Boban, Shereen Nabhani-Gebara

**Affiliations:** 1 School of Life Sciences, Pharmacy and Chemistry, Kingston University London, Penrhyn Road, Kingston Upon Thames, KT1 2EE UK; 2 Cyprus University of Technology, Limassol, Cyprus; 3 International Hellenic University, Thessaloniki, Greece; 4 Aristotle University of Thessaloniki, Thessaloniki, Greece; 5 Urology Department, Hospital Clinic de Barcelona, Barcelona, Spain; 6 Department of Advanced Biomedical Science, University of Naples Federico II, Naples, Italy; 7 Department of Radiology, Faculty of Medicine, University of Novi Sad, Hajduk Veljkova 3, 21000 Novi Sad, Serbia; 8 Diagnostic Imaging Center, Oncology Institute of Vojvodine, Put Dr Goldmana 4, 21204 Sremska Kamenica, Serbia; 9 University of Turku, Turku, Finland

**Keywords:** Artificial intelligence, Cancer care, Challenges, Experiences, Interviews, Healthcare professionals, Machine learning, Perceptions, Survey

## Abstract

**Background:**

The integration of Artificial Intelligence (AI) technology in cancer care has gained unprecedented global attention over the past few decades. This has impacted the way that cancer care is practiced and delivered across settings. The purpose of this study was to explore the perspectives and experiences of healthcare professionals (HCPs) on cancer treatment and the need for AI. This study is a part of the INCISIVE European Union H2020 project's development of user requirements, which aims to fully explore the potential of AI-based cancer imaging technologies.

**Methods:**

A mixed-methods research design was employed. HCPs participating in cancer care in the UK, Greece, Italy, Spain, Cyprus, and Serbia were first surveyed anonymously online. Twenty-seven HCPs then participated in semi-structured interviews. Appropriate statistical method was adopted to report the survey results by using SPSS. The interviews were audio recorded, verbatim transcribed, and then thematically analysed supported by NVIVO.

**Results:**

The survey drew responses from 95 HCPs. The occurrence of diagnostic delay was reported by 56% (n = 28/50) for breast cancer, 64% (n = 27/42) for lung cancer, 76% (n = 34/45) for colorectal cancer and 42% (n = 16/38) for prostate cancer. A proportion of participants reported the occurrence of false positives in the accuracy of the current imaging techniques used: 64% (n = 32/50) reported this for breast cancer, 60% (n = 25/42) for lung cancer, 51% (n = 23/45) for colorectal cancer and 45% (n = 17/38) for prostate cancer. All participants agreed that the use of technology would enhance the care pathway for cancer patients. Despite the positive perspectives toward AI, certain limitations were also recorded. The majority (73%) of respondents (n = 69/95) reported they had never utilised technology in the care pathway which necessitates the need for education and training in the qualitative finding; compared to 27% (n = 26/95) who had and were still using it. Most, 89% of respondents (n = 85/95) said they would be opened to providing AI-based services in the future to improve medical imaging for cancer care.

Interviews with HCPs revealed lack of widespread preparedness for AI in oncology, several barriers to introducing AI, and a need for education and training. Provision of AI training, increasing public awareness of AI, using evidence-based technology, and developing AI based interventions that will not replace HCPs were some of the recommendations.

**Conclusion:**

HCPs reported favourable opinions of AI-based cancer imaging technologies and noted a number of care pathway concerns where AI can be useful. For the future design and execution of the INCISIVE project and other comparable AI-based projects, the characteristics and recommendations offered in the current research can serve as a reference.

**Supplementary Information:**

The online version contains supplementary material available at 10.1186/s13014-023-02351-z.

## Introduction

Cancer remains a top cause of mortality and morbidity across the globe [1–4]. The latest statistics indicate an estimate of 10 million deaths and 19.3 million new cases in 2020 worldwide [2, 4, 5].These estimates are projected to increase substantially over the next two decades [2, 6] with an estimated increase of 47% in cancer incidence and 64% in cancer mortality by 2040 compared to 2020 [2, 6]. In Europe, an estimate of 2.7 million new cases and 1.3 million deaths were reported in 2020, highlighting the significant burden of the disease in the continent as well [3, 7]. Cancer is a complex and heterogenous disease that usually entails complex decision making, multiple handoffs between primary and specialty care providers, and active coordination amongst oncology team members [8]. Information technologies provide promise for cancer organisations to offer a quality patient-centred care delivery approach [8]. The deployment of new technologies in healthcare can promote patient care, improve patient outcomes, and promote workflow efficiencies through the greater use of automation, artificial intelligence (AI) and machine learning (ML) [9, 10]. AI has gained unprecedented attention over the past few decades and is currently considered the fourth industrial revolution [11]. The application of AI in cancer care has been rapidly emerging [12, 13]. Several applications for AI have been documented within the context of cancer care [9, 14, 15]. These include but are not limited to image contouring, image fusion and registration, treatment planning and quality assurance [9, 16–19]. While there is evidence supporting the positive effects of automation and AI in promoting the overall productivity and efficiency in oncology care [9], the perceptions and experiences of oncology-specialised healthcare professionals (HCPs) regarding cancer care and use of advanced technologies such as AI has not been widely considered to date. Hence, the current study aimed to address this issue and constituted a part of the user requirement definition of the INCISIVE project [20].

### An overview about the INCISIVE project

INCISIVE is a European Union (EU) Horizon 2020 funded project that brings together 26 industrial, clinical, and academic partners from across 9 European countries [21]. The project has two main aims. The first aim is to develop and validate an AI-toolbox to promote the cost-effectiveness of existing cancer imaging methods and performance of these methods in terms of accuracy, specificity, sensitivity, interpretability [20]. INCISIVE utilises data from the most common types of cancers: breast, prostate, lung, and colorectal cancer [5, 20]. The project will also deploy ML techniques by producing an automated ML-based annotation mechanism for medical images. The second aim is to develop an interoperable pan-European federated repository of health data including medical images. The ambition of INCISIVE is to build a repository that will enable the secure donation and sharing of data in accordance with ethical, legal and privacy demands. This in return can increase accessibility to datasets and enable the experimentation with AI-based solutions, thus contributing to the large-scale adoption of such solutions in cancer diagnosis, prediction, and follow-up [20].

However, the success of any technological interventions such as the one proposed in INCISIVE hinges on its acceptability and need among potential users [22, 23]. Hence, it is fundamental to explore the experiences and perceptions of HCPs in detail, being the primary users of the INCISIVE AI toolbox, regarding the gaps and challenges in current care, rooms for improvement in cancer imaging, experience with technology and the need for technology involving AI and ML such as the one proposed by INCISIVE.

## Materials and methods

### Study design

A mixed method research approach using a quantitative online survey and qualitative interviews was utilised to address the aims of this study.

### The quantitative phase: online survey with healthcare professionals

To ensure standardized reporting of the methods and results of the present online survey, researchers adopted the CHERRIES framework [24]. This framework is provided in Appendix 1 in Additional file 1.

#### Participants’ recruitment and data collection

Data collection was carried out using an anonymous online survey between February and April 2021. HCPs were recruited by INCISIVE partner countries/consortium: UK, Greece, Cyprus, Spain, Italy, and Serbia. The main inclusion criteria were: (i) be an HCP involved in lung, breast, colorectal or prostate cancer care and (ii) have good command of English. The questionnaire was administered using an online data collection methodology where a link to the questionnaire was created using Microsoft Forms. A participant information sheet (PIS) detailing the study and the questionnaire link were sent through email to the relevant partners in the consortium to send to eligible HCPs. The survey link was open for eight weeks to maximise response rate. Completeness checks were conducted after submitting the questionnaires. Respondents were able to review and change their answers via the back button up to the point of survey submission, as the survey was anonymous. Completion of the questionnaire was voluntary, and no incentives were provided to the participants.

#### Data collection tool

The questionnaire (Appendix 2 in Additional file 2) was designed by the research team based on the review of the relevant literature and other ongoing projects on the topic to address the study aims. Content validation was performed by sending the initial draft of the questionnaire to researchers and HCPs within the INCISIVE consortium to get an expert opinion about the instrument’s content, simplicity, relevance to the research topic and any other comments. The questionnaire consisted of 45 questions. Questions were predominantly closed ended of dichotomous style. The first section included 5 questions about demographics. The second section included 32 questions about the experience of HCPs with cancer care and challenges encountered. The section basically consisted of a set of 8 questions (6 closed ended and two open ended) that are repeatedly asked for each type of cancer, given that some HCPs are involved in the care of more than one tumour type. HCPs were only required to answer questions related to the tumour type they deal with in their practice. The third section included 7 questions in relation to technology use within the care pathway and willingness to use technology in future. The last section contained one free text question to provide any additional comments or suggestion in relation to the research topic. The questionnaire was designed to facilitate self-administration by the HCPs within an average of 15–25 min.

#### Sample size

This study was part of another study involving a discrete choice experiment (DCE). Hence, sample size calculation was based on a specific formula for DCE sample size calculation [25, 26].The formula indicated the need of 63 HCPs.

#### Data analysis

Data was imported from Microsoft Forms into the Statistical Package for Social Sciences (SPSS) software for analysis. Descriptive statistics were performed to describe participants’ characteristics and key findings using frequencies and percentages. The open-ended questions were analysed via content analysis [27].

### The qualitative phase: semi-structured interviews with healthcare professionals

#### Participants’ recruitment

A purposive sampling strategy based on the knowledge of the project’s consortium was utilised for participant’s recruitment across the same countries and using the same inclusion criteria as that used for survey recruitment.

#### Data collection tool

An interview topic schedule was developed based on the review of pertinent literature and other existing projects by the research team to guide and facilitate data collection. The interview schedule consisted of 20 questions (Appendix 3 in Additional file 3), encompassing aspects related to experience with the current care pathway, experience with technology use, and adoption of new technologies such as AI. At the end of the interview, the participants were asked to provide any additional information or comments that they feel important relevant to the discussed topic.

#### Sample size

Sampling was done iteratively until inductive thematic saturation was achieved, where no new information was emanating from the interviews. The stopping criterion indicating data saturation is three interviews which is the number of interviews performed without having new information, after which recruitment can be stopped [28]. Saturation occurred at the 24^th^ interview. Therefore, a subset of participants, totalling 27 healthcare professionals (HCPs), was recruited and interviewed, and all interviews were included in the final analysis.

#### Data collection

The interviews were conducted online via Microsoft Teams between January and April 2022 by two authors, DB and JK, a female and a male academic researcher. In total, 27 interviews were conducted with an average of 30 min (range: 20–35 min). No repeat interviews were conducted with any of the participants. The characteristics profile of participants is summarised in Table 1.

**Table 1:** Characteristics of HCPs participating in the interviews

Characteristic	Years	Number
Gender	Male	13
	Female	14
Years of experience in the oncology field	1–5	10
	6–10 years	9
	11–15 years	4
	16–20 years	1
	> 20 years	3
Country	Greece	6
	Italy	7
	Serbia	4
	Cyprus	8
	Spain	2
Occupation/ specialty	Medical Practitioner/ doctor	3
	Radiologist	4
	Medical Oncologist	2
	Radiation oncologist/ therapeutic radiographer/ radiotherapist	4
	Nuclear medicine physician	5
	Palliative care doctor/ rehabilitation doctor	2
	Surgeon/ Surgical oncologist	1
	Oncology nurse	4
	Urologist	2

#### Data analysis

Interviews were transcribed verbatim and subsequently analysed thematically using the five-stage framework approach [29, 30] by IH, DB and JK. Transcripts were not returned to participants for comments. The analysis was done in an iterative way where the first few interviews were transcribed and read to achieve data familiarisation and identify preliminary codes. Afterwards, each interview conducted was transcribed and coded to help guide with data saturation and hence recruitment. NVivo 12 software was used to facilitate transcripts coding. Transcripts’ coding and interpretation was a continuous and extensive process which involved reviewing, discussing, and checking the coded transcripts with the other co-authors. Any disagreements over data coding and interpretation were discussed between the co-authors until consensus was achieved. Codes were examined and then grouped into themes and subthemes. Derivation of themes was done using inductive and deductive approaches. All themes were given an equal weighting within the developed analytical framework. The final themes and subthemes were checked and verified by all authors to ensure analytical rigour and avoid bias in data analysis.

Each interviewee was assigned a pseudonym comprising of the participant’s number and the country name; for example, HCP1-Greece, with HCP standing as an acronym for healthcare professional. Data was presented in the form of themes and subthemes, with the use of direct quotations from participants to support findings under each theme/subtheme.

### Ethical considerations

Ethical approval was granted from the Research Ethics Committee at Kingston University on 29–01–2021 (reference No.2744). An electronic informed consent was acquired from HCPs willing to participate in the interviews. As for the survey, acceptance to complete and submit the questionnaire signified implied consent on the part of the participants.

## Results

### Survey with healthcare professionals

#### Response rate and participants demographics

A total of 95 HCPs completed the survey. Majority of the respondents were male (60%, n = 57/95). Nearly two third of respondents (62%, n = 59/95) were between ages 35 and 54 years and had more than 10 years of experience (65%, n = 62/95). HCPs from Greece (36%, n = 34/95) and Italy (26%, n = 26/95) constituted the majority of the respondents. Participants’ demographics are presented in Table 2.

**Table 2:** Characteristics of HCPs who participated in the survey

Participants’ characteristics	Number	Percentage
Gender		
Male	57	60
Female	38	40
Age		
< 25	0	0
25–34	20	21
35–44	30	32
45–54	29	30
55–64	15	16
≥ 65	1	1
Years of experience in the field of oncology		
< 1 year	2	2
1–5 years	18	19
6–10 years	13	14
11–15 years	27	28
16–20 years	10	11
> 20 years	25	26
Country		
UK	3	3
Serbia	9	10
Italy	25	26
Greece	34	36
Spain	17	18
Cyprus	7	7
Speciality/occupation		
General practitioner/doctor	6	6
Nurse	7	7
Pharmacist	4	4
Pathologist	2	2
Radiologist	12	13
Oncologist	12	13
Radiation oncologist/therapeutic radiographer/radiotherapist	7	7
Radiology technician	3	3
Nuclear medicine physician	12	13
Nuclear medicine technician	2	2
Urologist	12	13
Surgeon/ surgical oncologist	7	7
Other	9	10

### Healthcare professionals’ experience and perceptions of the four cancer care pathways

#### Experience and perceptions of the cancer care pathways

##### Breast cancer

About half of the HCPs (53%, n = 50/95) were involved in the care pathway of breast cancer. Participants were asked about the accuracy of the current imaging techniques used in breast cancer care in terms of false positives and false negatives. In this regard, 64% (n = 32/50) reported the occurrence of false positives. According to the respondents’ experience, the imaging techniques associated with false positives in order of frequency were mammography (n = 15), magnetic resonance imaging (MRI) (n = 13), PET/CT scan (n = 7), ultrasound (US) (n = 2), bone scan (n = 2) and computed tomography (CT) scan (n = 1). On the other hand, 50% (n = 25/50) reported the occurrence of false negatives. The imaging modalities associated with false negatives in order of frequency were mammography (n = 11), US (n = 9), PET/CT scan (n = 6), bone scan (n = 2) and MRI (n = 2).

Overall, 78% of HCPs (n = 39/50) perceived the current care pathway to be efficient. Nevertheless, more than half (56%, n = 28/50) indicated that patients face delays in diagnosis. Delay in referring patients to diagnostic tests/images was the main cited reason as indicated by 75% of HCPs (n = 21/28), followed by lack of adequate imaging resources/equipment (14%, n = 4/28), and lack of adequate staffing/understaffing (3.5%, n = 1/28). Furthermore, 40% (n = 20/50) indicated that patients face challenges particularly related to imaging tests, including: long waiting lists and delays in appointments (n = 9), lack of expertise and availability of imaging resources (n = 6), psychological distress due to false positives and false negatives (n = 2), and patients being reluctant to undergo diagnostic tests (n = 2).

##### Lung cancer

Just in excess of two fifths of the HCPs (44%, n = 42/95) were involved in the care pathway of lung cancer. Participants were also asked about the accuracy of the current imaging techniques for this cancer type in terms of false positives and false negatives. In this regard, 60% (n = 25/42) reported the occurrence of false positives. The imaging modalities associated with false positives in order of frequency were PET/CT scan (n = 16), followed by CT scan (n = 12). On the other hand, 57% (n = 24/42) indicated the occurrence of false negatives. The imaging techniques associated with false negatives in order of frequency were chest x-ray (n = 8), PET/CT scan (n = 5), followed by CT scan (n = 4).

Overall, 67% of HCPs (n = 28/42) perceived the current care pathway to be efficient. Nevertheless, nearly two-third (64%, n = 27/42) indicated that patients face delays in diagnosis. Delay in referring patients to diagnostic tests/images was the main cited reason (78%, n = 21/27), followed by lack of adequate staffing/understaffing (18.5%, n = 5/27). Additionally, 45% (n = 19/42) indicated that patients face challenges particularly related to imaging tests including: long waiting lists and delays in appointments (n = 10), lack of expertise (n = 4), lack of imaging resources (n = 5) and delays in imaging reporting (n = 2).

##### Colorectal cancer

Just less than half of the HCPs (47%, n = 45/95) were involved in the care pathway of colorectal cancer. Participants were also asked about the accuracy of the current imaging modalities in terms of false positives and false negatives. In this regard, 51% (n = 23/45) reported the occurrence of false positives. The imaging techniques associated with false positives in order of frequency were PET/CT scan (n = 14), CT scan (n = 6) followed by MRI (n = 5). On the other hand, 47% (n = 21/45) indicated the occurrence of false negatives. The imaging techniques associated with false negatives in order of frequency were CT scan (n = 8), MRI (n = 7), PET/CT scan (n = 3), followed by FDG PET/CT scan (n = 2) and colonoscopy (n = 2).

Overall, 56% of HCPs (n = 25/45) perceived the current care pathway to be efficient. More than three-quarters (76%, n = 34/45) indicated that patients face delays in diagnosis. Delay in referring patients to diagnostic tests/images was the main cited reason as indicated by 56% (n = 19/34), followed by lack of adequate imaging resources/equipment (21%, n = 7/34) and lack of adequate staffing/understaffing (15%, n = 5/34). In addition, 40% (n = 18/45) indicated that patients face challenges particularly related to imaging tests including: long waiting lists and delays in appointments (n = 5), lack of imaging resources (n = 5) and lack of expertise (n = 4).

##### Prostate cancer

Two fifths of the participants (40%, n = 38/95) were involved in the care of prostate cancer. Participants were asked if they have encountered any false positives and false negatives during the diagnostic procedure for prostate cancer. In this regard, 45% (n = 17/38) reported the occurrence of false positives. The imaging techniques/tests associated with false positives in order of frequency were prostate MRI (n = 4), PSMA PET/CT scan (n = 2), followed by choline PET/CT scan (n = 1) and PSA test (n = 1). On the other hand, 61% (n = 23/38) were aware of the occurrence of false negatives. The imaging techniques/tests associated with false negatives in order of frequency were biopsy (n = 4), MRI (n = 3), followed by FDG PET/CT scan (n = 1) and bone scan (n = 1).

Overall, 68% of HCPs (n = 26/38) perceived the current care pathway to be efficient. Less than half (42%, n = 16/38) indicated that patients face delays in diagnosis. Delay in referring patients to diagnostic tests/images was the main cited reason (56%, n = 9/16), followed by lack of adequate imaging resources /equipment (25%, n = 4/16) and lack of adequate staffing/understaffing (6%, n = 1/16). In addition, 39% (n = 15/38) indicated that patients face challenges particularly related to imaging tests, including: long waiting lists and delays in appointments (n = 4), lack of availability of imaging resources (n = 4).

### General challenges within the care pathway

HCPs were required to list three main challenges affecting the cancer care pathway, via an open-ended question. A total of 14 challenges were identified by content analysis across the 4 tumour types. The challenges were classified into 4 categories: (i) healthcare system related challenges, (ii) HCPs related challenges, (iii) clinical practice related challenges and (iv) patients related challenges. Across the four tumour types, the leading challenges were health care system related ones with the long waiting lists/times for diagnosis and treatment. The second challenge was lack of the different imaging modalities/tests within the institutions. Whereas in the third rank came the issue of lack of manpower/understaffing. The full list of challenges across the four tumour types is presented in Table 3.

**Table 3:** General challenges within breast, lung, colorectal and prostate cancer care pathways

Challenges within the care pathway of	Frequency for each tumour type				Total frequency across the 4 tumour types
	Breast cancer	Lung cancer	Colorectal cancer	Prostate cancer	Across the 4 tumour types
Healthcare system related challenges					
(1) Long waiting lists/times for diagnosis and treatment	22	27	25	9	83
(2) Lack of different imaging modalities/ tests	7	7	10	8	32
(3) Understaffing	9	8	7	5	29
(4) Lack of other resources	8	5	6	5	24
(5) Poor communication between different HCPs in the pathway	6	4	3	4	17
(6) Economic problems (financial cuts, lack of funding)	6	5	5		16
(7) Lack of multidisciplinary team (MDT) consensus	6	3	3	2	14
(8) Issues with accessibility: shortage of healthcare facilities/ departments specialised for breast cancer	3				3
(9) Poor organisation of the healthcare system	2				2
Healthcare professionals related challenges					
(10) Insufficient expertise	9	5	8	2	24
Clinical practice related challenges					
(11) Problems with the accuracy of imaging modalities		4	3	4	11
(12) Absence of national screening programme		2	2	2	6
Patient related challenges					
(13) Poor patient compliance to medical examination	3				3
(14) Financial problems/costs	2				2

### Challenges in cancer care that can be addressed using AI and ML

Using an open-ended question, HCPs were also required to list three main problems/challenges related to the use of imaging that could be resolved using AI and ML. A total of 10 main challenges were identified by content analysis across the 4 tumour types. The challenges were classified into 3 categories: (i) healthcare system related challenges, (ii) HCPs related challenges and (iii) clinical practice related challenges. The leading challenge that HCPs thought would be improved with AI and ML was clinical practice challenge related to the accuracy of the current imaging modalities/tests in terms of reporting and interpretation, and rates of false positive and false negative. The second was health care system related and constituted the reduction of long waiting lists/times for diagnosis and treatment, whereas the third was a clinical practice related challenges with disease evaluation in terms of characterisation, differentiation, and staging. The full list of challenges is presented in Table 4.

**Table 4:** Challenges that can be solved using AI and ML techniques across the four cancer types

Challenges that can be solved using AI and ML techniques	Frequency for each tumour type				Total frequency across the 4 tumour types
	Breast cancer	Lung cancer	Colorectal cancer	Prostate cancer	Across the 4 tumour types
Clinical practice related challenges					
(1) Problems with the accuracy of imaging modalities/tests: there is a need to improve the accuracy of current imaging modalities/tests in terms of reporting and interpretation, rates of false positive and false negative	22	17	17	11	67
(2) Challenges with disease evaluation: improvement in disease evaluation in terms of characterisation, differentiation and staging	5	13	10	4	32
(3) Insufficient standardisation of the care pathway: improvement in the standardisation of the care pathway	5	5	4	2	16
(4) Challenges with disease treatment: improvement in cancer treatment in terms of timing, choices and prognosis	2	4	4	2	12
(5) Challenges with disease recurrence: improve detection/prediction of cancer recurrence	2	2	2	4	10
Healthcare professionals related challenges					
(6) Insufficient expertise	4	4	5	2	15
(7) Human error: elimination of operator dependent error, reduction of interobserver/intraobserver variability	4			5	9
Healthcare system related challenges					
(8) Long waiting lists/times: reduction of long waiting lists/times for diagnosis and treatment	8	11	11	9	39
(9) Lack of resources: optimisation of resources (human, machinery and financial)	6	8	8	3	25
(10) Workload: reduction of workload	3				3

### Experience of technology use in oncology practice and acceptance of further technological interventions

All participants (n = 95) agreed that the use of technology would improve the care pathway for cancer patients. However, the majority (73%, n = 69/95) indicated no prior use of technology within the care pathway, versus 27% (n = 26/95) who have used technology and are still using it. Interestingly, all of those who are using technology but one (96%, n = 25/26) indicated that technology use is making a significant difference to patient’s care. The technologies currently used by some of the HCPs include Computer Aided Detection (CAD) and Picture Archiving and Communication System (PACS) systems.

The vast majority of respondents (89%, n = 85/95) indicated their willingness to deliver AI-based services to optimise medical imaging in cancer care in the future. Participants were asked via an open-ended question as to how an AI-based technology can gain the trust of HCPs and facilitate adoption. A total of 6 suggestions were identified by content analysis. The suggestions were classified into two main categories: (i) suggestions related to HCPs and (ii) suggestions related to the technology itself. The main and foremost suggestion was related to having an AI-technology that is evidence-based via randomised controlled trials (RCTs) to support its validity, reliability, and effectiveness. The second suggestion was the need to demonstrate the relative advantage of the AI-technology compared to current practice. The third was the provision of training and education. The fourth was pertinent to raising awareness about the technology among HCPs. Two suggestions were identified in the last rank, one was related to the fact that the AI-technology should not be perceived as replacement to the role of HCPs in decision making, and the other was the ease of use of the technology itself (Table 5).

**Table 5:** Suggestions provided by HCPs that can facilitate the adoption of an AI-technology and gain the trust of HCPs

Suggestions on how an AI-technology can gain the trust of HCPs and facilitate adoptions	Frequency
Suggestions related to technology	
(1) Evidence-based technology: the AI-technology needs to be evidence-based through robust research such as large RCTs to support the validity, reliability and effectiveness of the technology	29
(2) Relative advantage of the technology in comparison to current practice: the AI-technology should be more efficient compared to current traditional methods	7
(3) Ease of use: the technology should be easy to use and understand by the users (i.e., HCPs)	3
Suggestions related to healthcare professionals	
(4) Provision of training and education about AI to HCPs	5
(5) Raising awareness about the technology	4
(6) Technology not to be replacement of HCPs: the AI-technology should not be perceived as replacement to the role of HCPs	3

Using an open-ended question, participants were also required to elaborate on the elements/characteristics required in an AI tool in clinical practice. A total of six elements were identified. The first and foremost element was having an AI-tool that acts as a clinical decision support tool rather than a replacement for HCPs/medical expertise in clinical decisions (i.e. optimisation of decision making), so cross checking with expert advice needs to be available and the final decision needs to be for the HCPs. In the second rank, accuracy of the tool, and validity were chosen as important elements. Thirdly, ease of use, and reproducibility. Whereas the last element was related to AI explainability (Table 6).

**Table 6:** Elements an AI tool that would reinforce control in clinical practice as perceived by the participating HCPs

Elements of an AI tool that would reinforce HCPs’ feeling of being in control in clinical practice	Frequency
(1) The AI tool should perform as a clinical decision support tool rather than a replacement for HCPs in clinical decisions: the tool should provide suggestions rather than decisions with HCPs having the last word/say in clinical decisions	15
(2) Accuracy of the AI tool	5
(3) Validity: the evidence base behind the validity of the AI tool needs to be established	5
(4) Ease of use: the technology needs to be easy to use and understand by its users	3
(5) Reproducibility: the technology needs to generate reproducible results	3
(6) AI Explainability: the AI tool/system should be able to provide explanations about its decisions to the users (i.e., HCPs)	2

In addition, participants were asked about the best place within the care pathway for introducing an AI-based technology for optimisation of cancer imaging. Screening was chosen as the best place for the introduction of such technology by 40% (n = 37/95) followed by initial diagnosis (36%, n = 34/95) thereafter, further examination, disease staging and differentiation (16%, n = 15/95). Monitoring of treatment was the least favourable location for the introduction of an AI with only 9.5% of responses (n = 9/95).

### Interviews with healthcare professionals

Analysis of interviews revealed three main themes with associated subthemes.

#### Lack of widespread preparedness for AI in oncology

None of the respondents indicated prior or current use of AI in their clinical practice. Many HCPs perceived AI to be still in the initial or infancy phases with respect to oncology care. Hence, HCPs’ perceptions were divided between advocates and sceptics.

“I think we are in the very beginning, and I think we have to check the tool first, and we need experience before saying how confident I would feel about the tool.” (HCP8-Spain)

“…However, we are still in our infancy in the AI technologies.” (HCP10- Cyprus)

Some respondents advocated the introduction of AI via highlighting the potential benefits that AI would bring to oncology practice including: (1) aiding in clinical decision making, (2) promoting the efficiency of cancer care via making processes smoother and thus reducing the time spent across the different stages within the pathway, (3) reduction of interobserver variability, in addition to (4) reduction of clinicians’ workload via making tasks much quicker. The respondents reflected on several time-consuming tasks which they envisaged can be automated using AI such as tumour contouring, image segmentation, image quality checking, cases triaging and prioritisation.

“I think AI technology will play important role in diagnosis as it can speed up the process in diagnosis. Moreover, the AI tool will help doctors to contour the tumours more accurately…. It will help doctors to do their jobs faster, their workload is very high nowadays.” (HCP4- Greece).

“There are many advantages, more accurate diagnosis, significant reduction on time, reduction of delays in treatment and delay of therapy…. Interobserver variability may be reduced by AI…. (HCP7-Italy).

Whereas other HCPs were sceptical about AI introduction. Fear of jobs replacement by AI was raised by some respondents, which was intertwined with the issue of deskilling of HCPs as a result of over-reliance on AI in clinical practice. According to HCPs, losing clinical decision-making skills could lead to overlooking mistakes and errors that AI tools may produce, thus risking patients’ safety.

“…so the medical experience is gone, it would be gone in some years. You know when we extensively use AI and for a lot of medical professions like radiology, radiation oncology and so on. When we have just the AI doing the job, the physicians won't be able to do their jobs anymore of course, this is one problem, but on the other hand, who is checking the AI?’ (HCP18-Cyprus).

“There is a possibility for radiologists to lose their skills and ability to perform. Our capabilities on making difficult diagnoses might be affected because there is an AI that can do it for us.” (HCP5-Italy)

#### Barriers of AI in cancer care

Several barriers were articulated by HCPs regarding the introduction of AI in oncology care. Cost of implementation and infrastructure were identified as main barriers. Other barriers included: lack of HCPs’ time, age of HCPs as more senior colleagues might not be confident in using AI and could be sceptical as they are used to the more traditional ways of work, ethical issues surrounding data privacy and sharing, accountability in case of disagreement or when things go wrong in practice, lack of training and education in addition to fear of jobs replacement by AI.

“Well, it will be the funding. I think it's important. I think this might be another challenge, for example, to convince the people that they will not be replaced.” (HCP3-Greece)

“I can think of infrastructures right, because I mean, you would need access to this tool, so hardware everywhere, but also overlaps with the cost issue …based on my personal experience, many professional workers in healthcare are quite old. That's a gap that could be really hard to fill…. So, education, but just in point so if we do not want to wait for a generational change, then there’s a lot of teaching to do.” (HCP17- Italy).

“I think the major challenges to its confidentiality and where you're going to store all this data.” (HCP12- Cyprus)

“…to ensure privacy of data and to have legal and ethical clarity. And of course, there is the question if a diagnosis is wrong, who is responsible for this? Is it the AI algorithm, who wrote the algorithm or the doctor? Okay, there are such questions that are difficult to answer.” (HCP16- Greece).

Some respondents also cited patients’ perception of AI as a barrier. From HCPs’ perspective, lack of patients’ awareness about AI as a technology might cause agitation and disbelief, leading to a bad rapport between clinicians and patients. Thus, participants reported that increasing patient and public awareness of the advantages and benefits of AI in clinical practice is crucial for its effective implementation.

'At the beginning of the usage of AI, patients may be sceptical about whether these tools have some negative impact on their health…..' (HCP6-Serbia)

“…. I have read in some articles that patients do not trust an AI tool for their diagnosis…” (HCP16- Greece)

Additionally, the majority of the participants reported that explainability and interpretability are barriers to adopting AI in clinical practice. HCPs were against having an AI tool that functions as a black box. In addition, participants perceived that HCPs will only be able to cooperate with the novel AI technologies when they can understand how AI models work and what factors the AI models use to achieve clinical conclusions/decisions. Participants perceived that lack of transparency in the AI decision-making process could cause dilemmas and confusion among HCPs. Issues related to data availability, data quality and harmonisation also emerged as barriers.

“I will have the feeling of controlling the AI when I have the explanations of the parameters that the AI uses and to understand how AI is working or doing with the images, for example to have clear knowledge about the parameters used for analysis … I do not want to see it as a black box” (HCP4-Greece).

“I think the biggest challenge is to have a big sample of data, and the data to be harmonized and of quality….” (HCP26- Cyprus

#### Facilitators of AI in cancer care

Education and training of HCPs in addition to raising awareness among patients were depicted as crucial facilitators for AI implementation to alleviate any potential fear associated with the introduction of new technologies.

“…Patient awareness and training of healthcare professionals are important.” (HCP6-Serbia)

“I think there should be a training despite if it's easy, there should be a training because people sometimes are scared about the things they don't know.” (HCP20- Spain)

Having a tool that is both easy to use and user friendly, and evidence-based in terms of accuracy, reliability and validity also came as facilitators. The HCPs highlighted the need for a tool that will not require a lot of time and data input due to their immense workload.

“Reliability. If it's reliable then they will adopt it. If not then they will say I'm better than this so if it's reliable, if it shows that it can produce reliable results, then it will be adopted. Rigorous validation testing so they (referring to AI tools) would gain the trust.” (HCP1- Cyprus).

“…. I think first it should be user-friendly, obviously because everybody is very busy and the technology is advancing on the time, so it should be user friendly. (HCP12- Cyprus)

Time was also envisaged as an essential facilitator for AI implementation, as some respondents perceived that time is needed to allow HCPs to trial the AI tool in their institution and to adjust to experiencing it in order to see how it works and how they would incorporate it into their daily work routines.

“To work with the software for certain period as trial to practically its reliability. Not to be forced to use it. I would suggest internal trial for the software with all the doctors so then the tool can be validated in our clinical practice.” (HCP5- Italy).

“I think that everyone that has the AI tool wants time to play with the AI tool. To see the accuracy of the diagnosis, the sensitivity, the false negatives to see how it works and I think time is crucial for the implementation.” (HCP16- Greece).

## Discussion

The current paper provides a detailed investigation into HCPs’ perceptions, experiences and challenges in cancer care and needs for a technology involving AI such as the one proposed by the INCISIVE project. It also provides a guide for the design and implementation of the INCISIVE technology or any future AI interventions.

From the HCPs’ perspective, several challenges and gaps were identified within the care pathways for the four tumour types in general via the survey. Long waiting lists/delays in diagnosis and treatment was recognised as the leading challenge across the different countries for the four cancer types. Irrespective of the tumour type, HCPs indicated that patients face delays in diagnosis, particularly related to imaging and tests. Previous studies confirm the implications of delayed diagnosis where there’s a significant relationship between delay and increased mortality [31]. Delays experienced during diagnosis and treatment were also studied from a cancer survivor's perspective [32]. The delay in diagnosis increases the burden on both the patient and the health care system, which needs to be addressed through sustainable innovation models that could be achieved through automated technologies such as the one proposed by INCISIVE.

The challenges common across the four cancer types were: problems with the accuracy of the current imaging methods/tests, long waiting lists for diagnosis and treatment and lack of resources (human, machinery and financial). HCPs identified gaps in terms of the sensitivity (false negative) and specificity (false positive) rates of the different imaging modalities for the four cancer types, which in return would guide with the imaging modalities/tests that can be best optimised with the INCISIVE technology. The most common imaging modality/test associated with false positives was PET/CT scan for lung and colorectal cancer, mammography for breast cancer and MRI for prostate cancer. Whereas the most common imaging modality/test associated with false negatives was CT scan for colorectal cancer, chest x-ray for lung cancer, mammography for breast cancer and biopsy for prostate cancer.

HCPs also mentioned other important gaps that can be improved using Al and ML techniques, including cancer evaluation in terms of differentiation, characterisation and staging, in addition to cancer treatment in terms of timing, choices and prognosis, detection of cancer recurrence and standardisation of the current pathways. Addressing human errors was another gap that could be improved using AI and ML techniques by reducing operator dependent error and interobserver variability, as highlighted by the respondents.

AI and ML techniques are rapidly paving their way into cancer research and oncology care given their vast potential applications in this field [33]. AI has indeed shown great potential in several clinical settings within cancer care, including diagnosis, screening programs, disease monitoring, and recurrence detection. The application of AI in analyzing medical images such as X-rays, mammograms, and CT scans has generated promising results for early detection and classification of different types of cancers, such as breast cancer and lung cancer [34, 35].

In the context of cancer diagnosis, AI algorithms can help radiologists by analyzing medical images and identifying potential areas of concern. This can aid in the decision-making process and help prioritize high-risk cases. Likewise, AI-based tools can analyze chest X-rays or CT scans to identify suspicious lesions or nodules that may indicate lung cancer, thereby improving the accuracy and efficiency of cancer screening programs [36, 37].

AI's role in cancer care extends beyond diagnosis and screening. It can also contribute to monitoring cancer patients by analyzing varied data sources, including electronic health records, laboratory results, and imaging studies. Machine learning algorithms can identify trends, predict disease progression, and individualise treatment plans based on individual patient characteristics. Moreover, AI-powered tools can monitor treatment response and detect early signs of treatment side effects, allowing for appropriate interventions and improved patient outcomes [38, 39].

Another area where AI can make a major impact is in the detection of cancer recurrence. By comparing follow-up scans or surveillance imaging with baseline images, AI algorithms can identify subtle changes or new lesions that may show disease recurrence. Early detection of recurrence can aid to timely intervention and potentially improve patient outcomes [40, 41].

According to the survey, all HCPs agreed that the use of technology can improve the care pathway for cancer patients. This could be the reason that technology provides an opportunity to address certain unmet needs [42] and it is considered both a cure and a cause of global inequalities in cancer [43].

Despite that 73% of them indicated no prior use of technology in their clinical practice, the vast majority (89%) were very receptive to the concept of using AI and ML to optimise medical imaging in cancer care in the future, echoing previous literature [11, 44–46]. In terms of design and implementation, there was a clear preference among HCPs to have an intervention such as the one proposed by INCISIVE at the beginning of the journey, particularly at the stages of screening and initial diagnosis. This is due to the evidence linking late-stage cancer diagnosis with poor survival and avoidable deaths [47–50]. People diagnosed at an early stage are more likely to have better experience of oncology care, lower treatment morbidity, better survival and improved quality of life in comparison to those with late-stage diagnosis [31, 48–50]. The potential applications of AI in the oncology field are vast and promising, including detection and diagnosis of cancer, subtype classification, treatment optimisation and identification of new therapeutic targets in drug discovery [33]. An example of which is in a published study evaluating an AI-system in breast cancer based on screening mammography datasets from the UK and USA. The study showed an absolute reduction of 5.7% and 1.2% (USA and UK, respectively) in false positives and 9.4% and 2.7% in false negatives [36].

In order to remove prejudice within the HCP community, the following recommendations were identified. Having a technology that is evidence-based in terms of validity, reliability and effectiveness was the first and foremost recommendation provided by the HCPs. The second important recommendation is related to demonstrating to HCPs the additional benefits of the INCISIVE technology in comparison to the current methods they are using. Another recommendation was having a technology that can assist HCPs in their practice and not a technology that can/would potentially replace them. Threat to HCP identity was found across this study which was also identified by other studies published in the literature [46, 51, 52]. However, there is a controversy around this issue in the literature [9, 53]. A recent survey study among medical students and experienced physicians in Germany demonstrated how HCPs considered AI as a threat to both professional recognition and capabilities which contributed to resistance attitudes towards AI and its implementation in clinical practice [51]. Additionally, other articles stated how HCPs such as doctors, nurses and radiologists may perceive AI as a threat to their speciality with fears of job loss in the future [46, 52, 54]. This is also confirmed by a survey conducted in all 17 Canadian medical schools showing that 67% of medical students agreed that AI would reduce the demand for radiology and 48% were anxious for the future of the radiology speciality [55, 56]. Another international study by Huisman et al. [57] among 1041 radiologists and radiology residents from 54 countries reported mixed perceptions about AI in radiology. In the aforementioned research, although 48% of respondents had an open and proactive attitude towards AI, 38% reported fears of being replaced by AI. On the other hand, two studies revealed how the majority of their participants (doctors and medical students) did not believe that AI will replace them but rather revolutionise the medical field [45, 58]. In a third research, radiation oncologists believed that AI will most likely result in displacement of time-consuming repetitive tasks (contributing this way to the inefficiency problem within cancer care) rather than replacement of their roles [46]. This difference in opinion could stem from a difference in awareness and knowledge of AI.

Interviews with healthcare professionals revealed a lack of widespread preparedness for AI in oncology, barriers to introducing AI, and a need for education and training. Regardless of their development, AI applications in cancer are still considered to be in their infancy [18], which could explain lack of widespread preparedness to their adoption. However, some of the interview findings supported various benefits of AI [18, 60, 61]. This could be better understood in light of Rogers' theory on the diffusion of innovations (DOI), which details how new technical advancements travel across societies and cultures from inception to broad acceptance [62]. The five product characteristics (relative advantage, compatibility, complexity, observability, and trialability) that influence how attitudes are created [62] towards technology diffusion and it is observed in the present study. HCPs advocated for adopting AI as they deemed to make tedious tasks faster and promote the exactness of the results (relative advantage). There was also a clear need among the respondents for alternatives to reduce their workload which highlights the compatibility attribute in DOI. The need for lower level of complexity in AI innovation technologies was proposed to support users of all ages. This also necessitates the provisional periodic education and training for high-level complexity-related tasks. HCPs also highlighted the need for trusting AI (trialability) provided it can produce a valid result. More positive benefit (observability) about AI innovation bringing more accurate diagnosis was another element desired for AI use in clinical practice. All this implies that the introduction of AI is correlated with speeding up the clinical decision-making process, increasing access to cancer care, and improving clinical efficiency.

Even though AI is gaining traction in many fields, some of the barriers to AI adoption listed in the current study were economical (high cost of implementation), infrastructural (lack of physical structures and facilities), personal (lack of confidence in handling AI, unaccountability, and fear of job replacement), organisational (lack of HCPs’ time), ethical (issues with data privacy and sharing), and educational (lack of training) in nature. To successfully implement AI technology in cancer care, institutions must be equipped to address these challenges in a holistic manner. Strikingly, some of these barriers were also discovered in varied healthcare settings [63, 64]. The need for education and training was also highlighted as a crucial facilitator for AI implementation, which was also brought out by another study [13].

Another interesting and important point raised in the literature is the role of participants’ AI-specific knowledge as a predictor of AI fear. The research conducted by Huisman et al. [57] demonstrated that basic AI-specific knowledge was directly associated with fear, whereas intermediate and advanced AI-specific knowledge were inversely associated with fear from AI; hence, stressing the need to incorporate AI in medical training curricula especially in radiology to help facilitate its adoption in clinical practice [44], this was mirrored in another research which reported that tech-savvy medical students were less fearful of AI and more confident in its benefits and were advocate to the inclusion of AI in medical training [45]. Interestingly, in our study, provision of training and education and raising awareness about AI were another two valuable recommendations mentioned by the respondents that need to be considered in the design, validation and implementation phases of the INCISIVE project. A scoping review of the literature highlighted how lack of AI literacy can be a significant barrier to its adoption and use across all medical specialities. The review also recommended the adoption of competence-based curriculum design for AI in medical practice which should be spit across the 3 stages of medical education: undergraduate medical education, postgraduate medical education, and continuing professional development [52].

In conjunction to the above recommendations/suggestions, there were several elements/characteristics that were perceived by the HCPs to be essential to have in an AI-based technology to facilitate adoption. The main and foremost characteristic was having an AI tool that can act as a decision support tool rather than a replacement for HCPs in clinical decisions. The other essential elements were accuracy, validity, reproducibility, ease of use and explainability. Given the current findings, the mentioned characteristics should be also taken into consideration when designing the main features of the INCISIVE technology. It can be argued that these elements are of paramount importance to facilitate the adoption and implementation of the INCISIVE technology. According to the literature, an important yet understated obstacle to clinical implementation of AI is the frequent absence of user-friendly software to facilitate AI use in clinical institutions [33]. The implementation of AI must have its primary users in mind in order to be successful [33]. Another important issue which is getting more attention is explainability of AI [53], AI like any technology, has its own advantages and limitations. One of the main limitations of AI in radiology is the “black-box of AI” [41], which denotes to lack of interpretation of how AI works and how it arrives at its outcomes [54]. The significant potential of AI in oncology relies in situations where a clinical decision is otherwise challenging, possibly due to incomplete or conflicting observations between the different HCPs [33]. Therefore, for AI to help, it must have the ability/characteristic to explain its predictions and its decision-making process clearly so that users can gain confidence and trust in AI and are able to provide explanation of these predictions to colleagues and patients when needed [33]. Another important limitation in the field of AI is the unproven robustness of AI [33] or what is also termed as “AI chasm” which refers to the gap between the reported performance in laboratory conditions and performance in real-world context [54]. In clinical practice, AI models must stand up to a wide variety of fluctuations in data input, resolution, intensities and differences in disease features. However, a current problem is that most models are not tested enough to show robustness against such fluctuations or when tested, clearly demonstrate deterioration in performance [33]. Hence, AI models must be extensively tested and validated to achieve success in clinical institutions. In fact, there is a clear need for realistic evaluation of AI performance in real-world clinical settings through well-designed clinical trials rather than on a limited number of benchmark or challenge datasets [33, 67].

### Strengths and limitations

This study is among the few that explores the perceptions and experiences of multidisciplinary oncology-specialized healthcare professionals (HCPs) regarding cancer care and the use of advanced technologies like AI across various European countries. However, several limitations can be noted including the small sample size, which might have occurred due to several reasons including survey fatigue, the COVID-19 pandemic and lack of monetary incentives. Furthermore, the possibility of selection bias cannot be ruled out as participation might have occurred among HCPs who are more interested in AI as a topic. Despite these limitations, the present study reflected the opinions of different of oncology-specialised HCPs regarding cancer care and AI and provided an insightful guide to help the design and implementation of the INCISIVE project. It is important to note that the low enrolment ratio in our study restricts the representativeness and generalizability of the findings, primarily due to focusing on specific aspects of healthcare worker practices, language proficiency requirements, recruitment limited to six countries, a constrained data collection period, and enrolment determined by project requirements.

## Conclusion

Despite no prior use of AI in practice, oncology-specialised HCPs in the current study had positive attitudes towards AI-based technologies in cancer imaging and identified several challenges within the care pathway where AI can help. According to HCPs, AI holds the greatest potential at the stages of screening and diagnosis within the cancer care pathway. Several suggestions and characteristics were also provided in the current research which are important to help guide the design and implementation of the INCISIVE project and similar AI-based projects in the future.

### Supplementary Information

**Additional file 1**. Appendix 1: Checklist for Reporting Results of Internet E-Surveys (CHERRIES).

**Additional file 2**. Appendix 2: Online questionnaire for healthcare professionals.

**Additional file 3**. Appendix 3: Interview topic guide for healthcare professionals.

## Data Availability

The datasets used and/or analysed during the current study are available from the corresponding author on reasonable request.

## Abbreviations

AI: Artificial intelligence
CAD: Computer aided detection
CNS: Clinical specialist nurse
COVID-19: Coronavirus disease 19
CT: Computed tomography
DCE: Discrete choice experiment
DRE: Digital rectal examination
EBUS: Endobronchial ultrasound
EU: European Union
EUS: Endoscopic ultrasound
ESMO: European Society of Medical Oncology
ESTRO: European Society of Therapeutic Radiology and Oncology
FIT: Faecal immunochemical test
GOC: German Oncology Centre
GP: General practitioner
HCPs: Healthcare professionals
LDCT: Low dose computed tomography
MDT: Multidisciplinary team
ML: Machine learning
MR: Magnetic resonance
MRI: Magnetic resonance imaging
NCCN: National comprehensive cancer network
NHS: National Health Service
NICE: National Institute for Health and Care Excellence
PACS: Picture Archiving and Communication System
PET/CT: Positron emission tomography and computed tomography
PIS: Patient information sheet
PSA: Prostate specific antigen
RCT: Randomised controlled trial
TWW: Two-week wait
US: Ultrasound
UK: United Kingdom
