# Supplementary material for: Cancer care at the time of the fourth industrial revolution: an insight to healthcare professionals’ perspectives on cancer care and artificial intelligence
Source: Radiat Oncol. 2023 Oct 9;18:167. doi: 10.1186/s13014-023-02351-z (PMC10561443; doi:10.1186/s13014-023-02351-z)
Supplement: Supplementary file 1 — Additional file 1. Appendix 1: Checklist for Reporting Results of Internet E-Surveys (CHERRIES). [file 13014_2023_2351_MOESM1_ESM.docx]

**Appendix 1: Checklist for Reporting Results of Internet E-Surveys (CHERRIES)**

| **Item Category** | **Checklist Item** | **Explanation** | **Response** |
| --- | --- | --- | --- |
| Design | Describe survey design | Describe target population, sample frame. Is the sample a convenience sample? (In “open” surveys this is most likely.) | The target population for this study was healthcare professionals (HCPs) involved in lung, breast, colorectal, or prostate cancer care (4 types of cancer). The sample frame consisted of HCPs from the partner countries of the Incisive consortium, namely the UK, Greece, Cyprus, Spain, Italy, and Serbia. HCPs were recruited by the Incisive partner countries, indicating a convenience sampling approach based on the availability and willingness of HCPs from those countries to participate in the study. However, the inclusion criteria were specific to HCPs involved in cancer care and having a good command of English, which implies a purposive sampling strategy to target a specific group of HCPs with relevant expertise. |
| IRB (Institutional Review Board) approval and informed consent process | IRB approval | Mention whether the study has been approved by an IRB. | Ethical approval was granted from the Research Ethics Committee at Kingston University on 29-01-2021 (reference No.2744). |
|  | Informed consent | Describe the informed consent process. Were the participants told the length of time of the survey, which data were stored and where and for how long, who the investigator was, and the purpose of the study? | Participants were informed about various aspects of the study via the participant information sheet, including: the length of time required to complete the survey (15-25 minutes), the storage and location of the data (electronically stored at the university following security protocols for 10 years), the identity and affiliations of the investigators [principal investigator-SN, co-principal investigators-RK, and research associate-IH details were given for clarification] , and the purpose of the study (to explore the perspectives and experiences of healthcare professionals (HCPs) on cancer treatment and the need for AI. The survey was anonymous, hence, acceptance to complete and submit the questionnaire signified implied consent on the part of the participants. |
|  | Data protection | If any personal information was collected or stored, describe what mechanisms were used to protect unauthorized access. | No personal information was collected as the survey was anonymous. Information collected during the course of this study was collected and electronically stored at the university, following security protocols, for 10 years. The research team only had access to use the data in a way that prevented unauthorized access. This was highlighted/clarified in the participant information sheet. |
| Development and pre-testing | Development and testing | State how the survey was developed, including whether the usability and technical functionality of the electronic questionnaire had been tested before fielding the questionnaire. | The survey was developed by the research team, based on the review of the relevant literature, the project’s aims(s) and other ongoing projects on the topic to address the study aims. Content validation was performed by sending the initial draft of the questionnaire to researchers and HCPs within the INCISIVE consortium to get an expert opinion about the survey’s content, simplicity, relevance to the research topic, and any other comments. The questionnaire comprised 45 questions, with the majority of the questions being closed-ended and dichotomous in style. The technical functionality of the electronic questionnaire had been tested before fielding the questionnaire with few members at the university and no problems were identified. |
| Recruitment process and description of the sample having access to the questionnaire | Open survey versus closed survey | An “open survey” is a survey open for each visitor of a site, while a closed survey is only open to a sample which the investigator knows (password-protected survey). | Closed survey |
|  | Contact mode | Indicate whether or not the initial contact with the potential participants was made on the Internet. (Investigators may also send out questionnaires by mail and allow for Web-based data entry.) | The initial contact with the potential participants was made on the Internet via emails including a link to the survey and the participant information sheet and web-based data entry was allowed. |
|  | Advertising the survey | How/where was the survey announced or advertised? Some examples are offline media (newspapers), or online (mailing lists – If yes, which ones?) or banner Advertising the survey ads (Where were these banner ads posted and what did they look like?). It is important to know the wording of the announcement as it will heavily influence who chooses to participate. Ideally the survey announcement should be published as an appendix. | Participants were informed in advance through the Incisive consortium via an online mailing list. |
| Survey administration | Web/E-mail | State the type of e-survey (eg, one posted on a Web site, or one sent out through e-mail). If it is an e-mail survey, were the responses entered manually into a database, or was there an automatic method for capturing responses? | The survey was electronic with the survey link being created via Microsoft forms. An email about the survey was sent to healthcare professionals (HCPs) for self-administering the questions. The email included a link to the survey and the participant information sheet. The responses from the HCPs were recorded using Microsoft Forms. |
|  | Context | Describe the Web site (for mailing list/newsgroup) in which the survey was posted. What is the Web site about, who is visiting it, what are visitors normally Context looking for? Discuss to what degree the content of the Web site could pre-select the sample or influence the results. | The survey link was emailed to healthcare professionals (HCPs) from the partner countries of the INCISIVE consortium, namely the UK, Greece, Cyprus, Spain, Italy, and Serbia. The survey was not posted on any website. HCPs were directly contacted via email and provided with the survey link which was created via Microsoft forms. |
|  | Mandatory/voluntary | Was it a mandatory survey to be filled in by every visitor who wanted to enter the Web site, or was it a voluntary survey? | The survey was voluntary, and participants had the choice to participate in the survey or not. |
|  | Incentives | Were any incentives offered (eg, monetary, prizes, or non-monetary incentives such as an offer to provide the survey results)? | No monetary rewards, prizes, or non-monetary incentives were offered to participants for completing the survey. |
|  | Time/Date | In what timeframe were the data collected? | The data collection took place between February and April 2021. The survey link was open for eight weeks during this timeframe to maximize the response rate. |
|  | Randomization of items | To prevent biases items can be randomized or alternated. questionnaires | Items were not randomised in the survey. |
|  | Adaptive questioning | Use adaptive questioning (certain items, or only conditionally displayed based on responses to other items) to reduce number and complexity of the questions. | The research team did not receive any comments/requests to adapt any questions throughout the duration of the study. Besides, content validation was performed by sending the initial draft of the questionnaire to researchers and HCPs within the INCISIVE consortium to get an expert opinion about the questionnaire’s content, simplicity, relevance to the research topic and any other comments before enrolling the survey to the target population. |
|  | Number of Items | What was the number of questionnaire items per page? The number of items is an important factor for the completion rate. | The survey questionnaire consisted of 45 questions (Appendix 1). The average number of questions per page was 4-6 questions.  Questions were predominantly closed ended of dichotomous style. The first section included 5 questions about demographics. The second section included 32 questions about the experience of HCPs with cancer care and challenges encountered. The section basically consisted of a set of 8 questions (6 closed ended and two open ended) that are repeatedly asked for each type of cancer, given that some HCPs are involved in the care of more than one tumour type. The third section included 7 questions in relation to technology use within the care pathway and willingness to use technology in future. The last section contained one free text question to provide any additional comments or suggestion in relation to the research topic. |
|  | Number of screens (pages) | Over how many pages was the questionnaire distributed? The number of items is an important factor for the completion rate. | The survey questionnaire consisted of 12 pages. |
|  | Completeness check | It is technically possible to do consistency or completeness checks before the questionnaire is submitted. Was this done, and if “yes”, how (usually JAVAScript)? An alternative is to check for completeness after the questionnaire has been submitted (and highlight mandatory items). If this has been done, it should be reported. All items should provide a non-response option such as “not applicable” or “rather not say”, and selection of one response option should be enforced. | Completeness checks were conducted after submitting the questionnaires. When applicable, questions included an "other, please specify" option to accommodate additional responses. Furthermore, the questions were made mandatory, ensuring that respondents selected at least one response option. For instance, in the second section of the survey, healthcare professionals (HCPs) were presented with a repeated set of questions for each cancer type. If an HCP answered "yes" to the first question regarding involvement in the patient care pathway for a specific cancer type, the subsequent relevant questions related to that cancer type would appear. Conversely, if the HCP answered "no," they would be directed to the next cancer type. |
|  | Review step | State whether respondents were able to review and change their answers (eg, through a Back button or a Review step which displays a summary of the responses and asks the respondents if they are correct) | Respondents were able to review and change their answers via the back button up to the point of submission. After submitting the survey, participants were unable to change/modify or review answers as the survey was anonymous . |
| Response rates | Unique site visitor | If you provide view rates or participation rates, you need to define how you determined a unique visitor. There are different techniques available, based on IP addresses or cookies or both. | This was not provided/done. |
|  | View rate (Ratio of unique survey visitors/unique site visitor | Requires counting unique visitors to the first page of the survey, divided by the number of unique site visitors (not page views!). It is not unusual to have view rates of less than 0.1 % if the survey is voluntary | This was not measured as the survey was created via Microsoft forms and not added on a website. |
|  | Participation rate (Ratio of unique visitors who agreed to participate/unique first survey page visitors) | Count the unique number of people who filled in the first survey page (or agreed to participate, for example by checking a checkbox), divided by visitors who visit the first page of the survey (or the informed consents page, if present). This can also be called “recruitment” rate. | It was not feasible to measure the participation rate as there was not a separate informed consent form given that the survey was anonymous. So, in this study, responses were anonymous and acceptance to complete and submit the questionnaire signified implied consent on the part of the participants. There was no log for participants who only agreed to participate or filled first page of the survey. The log provided by Microsoft forms only showed participants who submitted the questionnaire. |
|  | Completion rate (Ratio of users who finished the survey/users who agreed to participate) | The number of people submitting the last questionnaire page, divided by the number of people who agreed to participate (or submitted the first survey page). This is only relevant if there is a separate “informed consent” page or if the survey goes over several pages. This is a measure for attrition. Note that “completion” can involve leaving questionnaire items blank. This is not a measure for how completely questionnaires were filled in. (If you need a measure for this, use the word “completeness rate”.) | It was not feasible to measure the completion rate as there was not a separate informed consent form given that the survey was anonymous. So, in this study, responses were anonymous and acceptance to complete and submit the questionnaire signified implied consent on the part of the participants. There was no log for participants who only agreed to participate. The log provided by Microsoft forms only showed participants who submitted the questionnaire. |
| Preventing multiple entries from the same individual | Cookies used | Indicate whether cookies were used to assign a unique user identifier to each client computer. If so, mention the page on which the cookie was set and read, and how long the cookie was valid. Were duplicate entries avoided by preventing users access to the survey twice; or were duplicate database entries having the same user ID eliminated before analysis? In the latter case, which entries were kept for analysis (eg, the first entry or the most recent)? | Cookies were not used. Nevertheless, Participants were unable to modify their responses or resubmit once the survey has been submitted as the survey was only displayed once for each participant. Additionally, given the busy work environment of HCPs in the oncology field, it is unlikely that participants had duplicates. |
|  | IP check | Indicate whether the IP address of the client computer was used to identify potential duplicate entries from the same user. If so, mention the period of time for which no two entries from the same IP address were allowed (eg, 24 hours). Were duplicate entries avoided by preventing users with the same IP address access to the survey twice; or were duplicate database entries having the same IP address within a given period of time eliminated before analysis? If the latter, which entries were kept for analysis (eg, the first entry or the most recent)? | This was not done. Nevertheless, Participants were unable to modify their responses or resubmit once the survey has been submitted as the survey was only displayed once for each participant. Additionally, given the busy work environment of HCPs in the oncology field, it is unlikely that participants had duplicates. |
|  | Log file analysis | Indicate whether other techniques to analyze the log file for identification of multiple entries were used. If so, please describe. | This was not done. Nevertheless, the participants were clearly instructed to fill the questionnaire only once. Additionally, given the busy work environment of HCPs in the oncology field, it is unlikely that participants had duplicates. |
|  | Registration | In “closed” (non-open) surveys, users need to login first and it is easier to prevent duplicate entries from the same user. Describe how this was done. For example, was the survey never displayed a second time once the user had filled it in, or was the username stored together with the survey results and later eliminated? If the latter, which entries were kept for analysis (eg, the first entry or the most recent)? | In the current study, participants were unable to modify their responses or resubmit once the survey has been submitted as the survey was only displayed once for each participant. |
| Analysis | Handling of incomplete questionnaires | Were only completed questionnaires analyzed? Were questionnaires which terminated early (where, for example, users did not go through all questionnaire pages) also analyzed? | There was a great emphasis from the project consortium to encourage participants to provide complete questionnaires. Hence, all questionnaires received were complete with no missing data/ questions. |
|  | Questionnaires submitted with an atypical timestamp | Some investigators may measure the time people needed to fill in a questionnaire and exclude questionnaires that were submitted too soon. Specify the timeframe that was used as a cut-off point and describe how this point was determined. | All questionnaires were submitted within the allocated timeframe as anticipated. Average timestamp of completion was 15-35 minutes (average time to complete the questionnaire on Microsoft forms). This average timeframe was based on the content validation which was done to validate the content of the questionnaire and its simplicity before enrolling the questionnaire to the participants. |
|  | Statistical correction | Indicate whether any methods such as weighting of items or propensity scores have been used to adjust for the non-representative sample; if so, please describe the methods | This was not done as the survey was descriptive in nature. |
