# Supplementary material for: Cancer care at the time of the fourth industrial revolution: an insight to healthcare professionals’ perspectives on cancer care and artificial intelligence
Source: Radiat Oncol. 2023 Oct 9;18:167. doi: 10.1186/s13014-023-02351-z (PMC10561443; doi:10.1186/s13014-023-02351-z)
Supplement: Supplementary file 2 — Additional file 2. Appendix 2: Online questionnaire for healthcare professionals. [file 13014_2023_2351_MOESM2_ESM.docx]

**Appendix 2: Online questionnaire for healthcare professionals**

**Experience and perceptions of cancer care across Europe**

This form contains a list of questions about some socio-demographic information and other questions about the care you provide to cancer patients. Please take few minutes to answer all of them. Your responses will be anonymous and entirely confidential and will not be used for any purpose other than research for the INCISIVE project. Please, answer by ticking the relevant box, circling the appropriate answer or filling the space provided.

**Online informed consent**

Do you consent to participate and fill the questionnaire?

Yes (If yes, participant will proceed to the questionnaire)

No (if No, participant will proceed to the study debrief section)

**Section 1: Demographics and general information**

1. Gender:

Male

Female

Prefer not to answer

1. Age:

Under 25

25-34

35-44

45-54

55-64

≥ 65

1. Job title in your current organisation

General Practitioner (GP)/ doctor

Nurse

Pharmacist

Pathologist

Oncologist

Radiation oncologist / therapeutic radiographer

Radiologist

Radiotherapist

Radiology technician

Nuclear medicine physician

Nuclear medicine technician

Palliative care doctor

Other (please, specify) --------------------------------------------------------------------------------------------------------------------------------------------------------------------------------------------------------------------------------------------------------

1. How many years of experience do you have?

Less than one year

1-5 years

6-10 years

11-15 years

16-20 years

> 20 years

1. Country of work/employment

England

Italy

Greece

Serbia

Spain

Finland

Cyprus

**Section 2: Experience and perception of the care pathway**

1. Are you involved in the patient care pathway for **LUNG CANCER**?

Yes

No

1. Considering the accuracy of the current imaging techniques used in your organisation, are you aware of any false positives that occur?

Yes

No

If, yes, please explain which technique and how often does that happen?------------------------------------------------------------------------------------------------------------------------------------------------------------------------------------------------------------------------------------------------------------------------------------------------

1. Considering the accuracy of the current imaging techniques used in your organisation, are you aware of any false negatives that occur?

Yes

No

If yes, please explain which technique and how often does that happen?------------------------------------------------------------------------------------------------------------------------------------------------------------------------------------------------------------------------------------------------------------------------------------------------

1. In your experience, do patients in this pathway face any delays in diagnosis (Long waiting time between reported symptoms and diagnosis)?

Yes

No

If yes, what is the main reason for the delay?

Patients not referred quickly to diagnostic tests/images

Lack of adequate imaging resources/equipment

Lack of adequate staffing/understaffing

Other, please specify----------------------------------------------------------------------------------------------------------------------------------------------------------------------------------------------------------------------------------------------------

1. In your experience, do patients in this pathway face any challenges related to imaging tests?

Yes

No

If yes, please specify, -------------------------------------------------------------------------------------------------------------------------------------------------------------------------------------------------------------------------------------------------------------------------------------------------------------------------------------------------------------------------------------

1. List the 3 main challenges affecting the care pathway in general

------------------------------------------------------------------------------------------------------------------------------------------------------------------------------------------------------------------------------------------------------------------------------------------------------------------------------------------------------------------------------------------------------------------------------------------------------------------------------------------------------------------------------

1. List the 3 main problems/challenges related with the use of imaging in cancer care that in your opinion could be resolved using machine learning and artificial intelligence techniques.

------------------------------------------------------------------------------------------------------------------------------------------------------------------------------------------------------------------------------------------------------------------------------------------------------------------------------------------------------------------------------------------------------------------------------------------------------------------------------------------------------------------------------

1. Overall, do you think the current care pathway is efficient?

Yes

No

Why?--------------------------------------------------------------------------------------------------------------------------------------------------------------------------------------------------------------------------------------------------------------------------------------------------------------------------------------------------------------------------------------------------

1. Are you involved in the patient care pathway for **BREAST CANCER**?

Yes

No

1. Considering the accuracy of the current imaging techniques used in your organisation, are you aware of any false positives that occur?

Yes

No

If yes, please explain which technique and how often does that happen?----------------------------------------------------------------------------------------------------------------------------------------------------------------------------------------------------------------------------------------------------------------------------------------------------------------

1. Considering the accuracy of the current imaging techniques used in your organisation, are you aware of any false negatives that occur?

Yes

No

If yes, please explain which technique and how often does that happen?------------------------------------------------------------------------------------------------------------------------------------------------------------------------------------------------------------------------------------------------------------------------------------------------

1. In your experience, do patients in this pathway face any delays in diagnosis (Long waiting time between reported symptoms and diagnosis)?

Yes

No

If yes, what is the main reason for the delay?

Patients not referred quickly to diagnostic tests/images

Lack of adequate imaging resources/ equipment

Lack of adequate staffing /understaffing

Other, please specify-------------------------------------------------------------------------------------------------------------------------------------------------------------------------------------------------------------

1. In your experience, do patients in this pathway face any challenges related to imaging tests?

Yes

No

If yes, please specify, -----------------------------------------------------------------------------------------------------------------------------------------------------------------------------------------------------------------------------------------------

1. List the 3 main challenges affecting the care pathway

------------------------------------------------------------------------------------------------------------------------------------------------------------------------------------------------------------------------------------------------------------------------------------------------------------------------------------------------------------------------------------------------------------------------------------------------------------------------------------------------------------------------------

1. List the 3 main problems/challenges related with the use of imaging in cancer care that in your opinion could be resolved using machine learning and artificial intelligence techniques.

------------------------------------------------------------------------------------------------------------------------------------------------------------------------------------------------------------------------------------------------------------------------------------------------------------------------------------------------------------------------------------------------------------------------------------------------------------------------------------------------------------------------------

1. Overall, do you think the current care pathway is efficient?

Yes

No

Why?---------------------------------------------------------------------------------------------------------------------------------------------------------------------------------------------

1. Are you involved in the patient care pathway for **COLORECTAL CANCER**?

Yes

No

1. Considering the accuracy of the current imaging techniques used in your organisation, are you aware of any false positives that occur?

Yes

No

Please explain which technique and how often does that happen?------------------------------------------------------------------------------------------------------------------------------------------------------------------------------------------------------------------------------------------------------------------------------------------------------------------------

1. Considering the accuracy of the current imaging techniques used in your organisation, are you aware of any false negatives that occur?

Yes

No

Please explain which technique and how often does that happen?------------------------------------------------------------------------------------------------------------------------------------------------------------------------------------------------------------------------------------------------------------------------------------------------------------------------

1. In your experience, do patients in this pathway face any delays in diagnosis (Long waiting time between reported symptoms and diagnosis)?

Yes

No

If yes, what is the main reason for the delay?

Patients not referred quickly to diagnostic tests/images

Lack of adequate imaging resources/ equipment

Lack of adequate staffing /understaffing

Other, please specify----------------------------------------------------------------------------------------------------------------------------------------------------------------------------------------------------------------------------------------------------

1. In your experience, do patients in this pathway face any challenges related to imaging tests?

Yes

No

If yes, please specify, ----------------------------------------------------------------------------------------------------------------------------------------------------------------------------------------------------------------------------------------------------------------------------------------

1. List the 3 main challenges affecting the care pathway in general

------------------------------------------------------------------------------------------------------------------------------------------------------------------------------------------------------------------------------------------------------------------------------------------------------------------------------------------------------------------------------------------------------------------------------------------------------------------------------------------------------------------------------

1. List the 3 main problems/challenges related to the use of imaging in cancer care that in your opinion could be resolved using machine learning and artificial intelligence techniques.

------------------------------------------------------------------------------------------------------------------------------------------------------------------------------------------------------------------------------------------------------------------------------------------------------------------------------------------------------------------------------------------------------------------------------------------------------------------------------------------------------------------------------

1. Overall, do you think the current care pathway is efficient?

Yes

No

Why?-------------------------------------------------------------------------------------------------------------------------------------------------------------------------------------------------------------------------------------------------------------------------------------------------------------------------------------------------------------------------------------------------

1. Are you involved in the patient care pathway for **PROSTATE CANCER**?

Yes

No

1. With respect to diagnosis, have you encountered any false positives?

Yes

No

If yes, please explain how and why?----------------------------------------------------------------------------------------------------------------------------------------------------------------------------------------------------------------------------------------------------------

1. With respect to diagnosis, have you encountered any false negatives?

Yes

No

If yes, please explain how and why?-----------------------------------------------------------------------------------------------------------------------------------------------------------------------------------------------------------------------------------------------------

1. In your experience, do patients in this pathway face any delays in diagnosis (Long waiting time between reported symptoms and diagnosis)?

Yes

No

If yes, what is the main reason for the delay?

Patients not referred quickly to diagnostic tests/images

Lack of adequate imaging resources/ equipment

Lack of adequate staffing /understaffing

Other, please specify----------------------------------------------------------------------------------------------------------------------------------------------------------------------------------------------------------------------------------------------------

1. In your experience, do patients in this pathway face any challenges related to imaging tests?

Yes

No

If yes, please specify, ----------------------------------------------------------------------------------------------------------------------------------------------------------------------------------------------------------------------------------------------------------------------------------------

1. List the 3 main challenges affecting the care pathway in general

------------------------------------------------------------------------------------------------------------------------------------------------------------------------------------------------------------------------------------------------------------------------------------------------------------------------------------------------------------------------------------------------------------------------

1. List the 3 main problems/challenges related to the use of imaging in cancer care that in your opinion could be resolved using machine learning and artificial intelligence techniques.

------------------------------------------------------------------------------------------------------------------------------------------------------------------------------------------------------------------------------------------------------------------------------------------------------------------------------------------------------------------------------------------------------------------------

1. Overall, do you think the current care pathway is efficient?

Yes

No

Why?-----------------------------------------------------------------------------------------------------------------------------------------------------------------------------------------------------------------------------------------------------------------------------------------------

**Section 3: Experience of technology and acceptance of further technological interventions**

1. Do you think the use of technology can improve the care pathway for cancer patients?

Yes

No

Why?-----------------------------------------------------------------------------------------------------------------------------------------------------------------------------------------------------------------------------------------------------------------------------------------------

1. Have you used any technology-based service as part of the care pathway for cancer patients?

Yes

No

If yes, what was the service ?------------------------------------------------------------------------------------------------------------------------------------------------------------------------------------------------------------------------------------------------------------------

1. Are you currently using any technology-based services as part of the care pathway?

Yes

No

If yes, what is it ?---------------------------------------------------------------------------------------------------------------------------------------------------------------------------------------------------------------------------------------------------------------------------------

If yes, do you think the service is making a significant difference to patient care?

Yes

No

1. Are you willing to deliver a technology-based service which involves Artificial Intelligence (AI) to optimise medical imaging in cancer care in the future?

Yes

No

Why?-------------------------------------------------------------------------------------------------------------------------------------------------------------------------------------------------------------------------------------------------------------------------------------------------------------------------------------------------------------------------------------------------

1. If you are provided with an AI technology that would optimise medical imaging in cancer care, where do you think the best place for this technology would be in the care pathway?

Screening

Initial diagnosis

Further examination/ Disease staging and differentiation

Monitoring of treatment

1. How do you think a proposed AI-based technology can gain the trust of healthcare professionals and facilitate adoption?

------------------------------------------------------------------------------------------------------------------------------------------------------------------------------------------------------------------------------------------------------------------------------------------------------------------------------------------------------------------------------------------------

1. Which elements of an AI tool in clinical practice would reinforce your feeling of being in control?

------------------------------------------------------------------------------------------------------------------------------------------------------------------------------------------------------------------------------------------------------------------------------------------------------------------------------------------------------------------------------------------------------------------

**Section 4: Additional information**

1. Please provide any further comments you want to share with us

------------------------------------------------------------------------------------------------------------------------------------------------------------------------------------------------------------------------------------------------------------------------------------------------------------------------------------------------------------------------------------------------------------------------------------------------------------------------------------------------------------------------------
