# Supplementary material for: Cancer care at the time of the fourth industrial revolution: an insight to healthcare professionals’ perspectives on cancer care and artificial intelligence
Source: Radiat Oncol. 2023 Oct 9;18:167. doi: 10.1186/s13014-023-02351-z (PMC10561443; doi:10.1186/s13014-023-02351-z)
Supplement: Supplementary file 3 — Additional file 3. Appendix 3: Interview topic guide for healthcare professionals. [file 13014_2023_2351_MOESM3_ESM.docx]

**Appendix 3: Interview topic guide for healthcare professionals**

**Section 1: Experience of the current care pathway**

1. Can you provide me with an overview about your experience with care for cancer patients/survivors?
2. What are the challenges that you have encountered whilst caring for cancer patients?

Prompt:

Enquire whether the challenges are related to patients, work environment and other healthcare professionals, the care pathway or other.

Enquire about any delays encountered by the patients.

1. In your opinion, where is the bottleneck within the care pathway? Why?

Prompt: phases of the pathway: diagnosis, treatment, monitoring and follow-up

1. What are the main problems/ challenges in diagnostics, treatment definition and follow-up that relate to imaging data that in your opinion could be resolved using Machine Learning (ML) and Artificial Intelligence (AI) techniques?
2. Have you encountered any false negatives (patient first cleared but turned out to have cancer later)? Can you please elaborate with examples?
3. Have you encountered any false positives? Can you please elaborate with examples?
4. In your opinion, how can the care pathway for cancer patients be improved?

**Section 2: Experience with technology use**

1. Have you used any technology-based services as part of the care pathway?

Prompt:

If No, elaborate on the reason

If Yes, elaborate on the service used and whether it was beneficial to the participant and their patients.

1. Are you currently using any technology-based services as part of the care pathway?

Prompt:

If No, elaborate on the reason

If Yes, elaborate on the service used and whether it is beneficial to the participant and their patients.

**At this stage, the interviewer will explain to the participant about the INCISIVE Artificial Intelligence (AI) platform and proceed with the below questions:**

1. What do you think about the introduction of the INCISIVE AI platform in the care pathway for cancer patients in the future? Why?

Prompt: investigate about the potential of the INCISIVE technology in helping with the challenges related to the use of imaging.

1. In your opinion, where do you see the best place for the INCISIVE AI platform in the care pathway (i.e. at which stage in the care pathway)? Why?

Prompt: elaborate on whether the participant sees the best place for the AI platform upon initial diagnosis, further examination/ Disease staging and differentiation or upon monitoring and follow-up.

1. Will you be opt to deliver a technology based service like the INCISIVE AI platform to aid in diagnosis and monitoring of your patients in the future? Why?
2. How confident do you feel about delivering a service involving Information Technology, particularly artificial intelligence (AI) in order to support your patients?
3. In your opinion, what are the most important factors that would facilitate the use and adoption of an AI-based technology?
4. How do you think a proposed AI-based technology can gain the trust of healthcare professionals and facilitate adoption?
5. In your opinion, what are the barriers/challenges that would affect the use and adoption of an AI-based technology?
6. Which elements of an AI tool in clinical practice would reinforce your feeling of being in control”?
7. What do you think are the advantages and disadvantages of using a technology like the INCISIVE AI platform?

**Section 3: Adoption of innovations in the country**

1. Who are the decision makers involved in investing in new technologies like the INCISIVE AI platform in your organisation?
2. Who are the influencers involved in the investment process for new technologies like the INCISIVE AI platform in your organisation?

**Further additions/ comments**

Do you have any questions that you would like to ask me?

Is there anything else you would like to share or add about this subject?
